# Effect of 1% sodium alendronate in the non-surgical treatment of periodontal intraosseous defects: a 6-month clinical trial

**DOI:** 10.1590/1678-7757-2016-0252

**Published:** 2017

**Authors:** Bernardo Carvalho DUTRA, Alcione Maria Soares Dutra OLIVEIRA, Peterson Antônio Dutra OLIVEIRA, Flavio Ricardo MANZI, Sheila Cavalca CORTELLI, Luís Otávio de Miranda COTA, Fernando Oliveira COSTA

**Affiliations:** 1 Universidade Federal de Minas Gerais, Faculdade de Odontologia, Departamento de Patologia e Cirurgia. Belo Horizonte, MG, Brasil.; 2 Pontífica Universidade Católica of Minas Gerais, Departamento de Periodontia. Belo Horizonte, MG, Brasil.; 3 Universidade de Taubaté, Departamento de Odontologia, Divisão de Pesquisa Periodontal. Taubaté, SP, Brasil.

**Keywords:** Sodium, alendronate, Biphosphonates, Periodontitis, Dental scaling

## Abstract

**Background and objectives:**

Few studies have evaluated the effect of the topical application of sodium alendronate (ALN) on the treatment of intrabuccal bone defects, especially those caused by periodontitis. This 6-month randomized placebo controlled clinical trial aimed at evaluating the effect of non-surgical periodontal treatment associated with the use of 1% ALN, through clinical evaluations and cone-beam computed tomography (CBCT).

**Material and Methods:**

Twenty individuals with chronic periodontitis underwent periodontal examination at the baseline as well as 3 and 6 months after periodontal treatment, registering clinical attachment level (CAL), periodontal probing depth (PPD), and bleeding on probing (BOP) as the clinical outcomes. After manual scaling and root planing, 40 bilateral sites with interproximal vertical bone defects were randomly treated with either 1% ALN gel or a placebo. Bone defects were evaluated through CBCT at the baseline and 6 months post-treatment. The clinical and CBCT parameters were compared using the Wilcoxon and Friedman tests (p<0.05).

**Results:**

Although ALN produced a greater CAL gain when compared to the placebo at 6 months post-treatment (p=0.021), both treatments produced similar effects on the PPD, BOP, and bone height. Significant differences in bone fill were observed only in patients of the ALN group (4.5 to 3.8 mm; p=0.003) at 6 months post-treatment.

**Conclusions:**

Topical application of 1% ALN might be a beneficial adjuvant to non-surgical periodontal therapy.

## Introduction

Chronic periodontitis is deﬁned as inﬂammation of the gingiva extending into the adjacent attachment apparatus^3^. The disease is characterized by loss of clinical attachment due to the destruction of the periodontal ligament and of the adjacent supporting bone. It is highly prevalent among adult populations^1^. Because of the previously demonstrated capacity of the periodontal tissues for regeneration^8^, there has been increased interest in the mechanisms of periodontal wound healing and the biological factors and cells involved in this process^13^. A wide range of regenerative techniques has been proposed along the years, many of those involving bone grafts or their synthetic substitutes^13^, guided tissue regeneration^21^, topical application of growth factors^22^, stem cells^11^, antiresorptive agents^27^, or combinations of procedures^13,22^. However, some of these regenerative techniques have restricted predictability, high cost, and technical difficulties. Currently, the delivery of bioactive molecules using vehicles known as carrier agents has an important role in tissue regenerative therapy^7^.

Biphosphonates (BPhs) are a group of bone metabolism mediators, presently used to treat osteoclast-mediated bone loss due to osteoporosis and other conditions^5,9^. Due to their effects on the differentiation, activity, and apoptosis of osteoclasts, they slow down the rate of bone loss and increase the resistance to bone fracture^19^. Sodium alendronate (ALN), an aminobisphosphonate, acts as a potent inhibitor of bone resorption^4,10^. Due to the high affinity of ALN for hydroxyapatite, it has been suggested that the ALN drug complex is retained at the site of injection^12,16^. *In vitro* studies demonstrated that low-dose ALN was effective in inhibiting osteoclastic activity with no cytotoxic effects^20^, as well as effect on regulatory genes of osteoblasts differentiation^2,10,14^Therefore, some studies have been conducted to evaluate the efficacy of ALN in the treatment of bone and periodontal disorders^26-28,33^. Animal studies have evaluated the effects of both the topical^6,10,16^and systemic^15,17,32^use of ALN in the treatment of intrabuccal bone defects resulting from periodontitis. In humans, clinical trials have investigated the effects of the topical application of ALN on periodontal bone defects treated with surgical^27^ or non-surgical procedures^17,26,28,29^. It is important to note that no adverse effects were reported in any study on the topical application of ALN^26,28,29.^

While the majority of the studies were designed to evaluate the effects of 1% ALN gel on bone parameters, only few studies focused on its effects on periodontal clinical parameter^25^. Although improvements in periodontal probing depth (PPD), clinical attachment level (CAL), bleeding on probing (BOP), and gingival index (GI) resulting from the use of ALN have already been reported^17,26-29^, the available data is still controversial. Therefore, further studies are required for a better understanding on how ALN affects periodontal healing in periodontitis patients.

Based on the hypothesis that the adjunctive use of ALN would improve periodontal status, this 6-month randomized placebo controlled clinical trial evaluated the effect of scaling and root planing in combination with the topical application of 1% ALN gel on periodontal clinical parameters and cone-beam computed tomography (CBCT) measurements.

## Material and methods

### Study population

**Figure 1:**
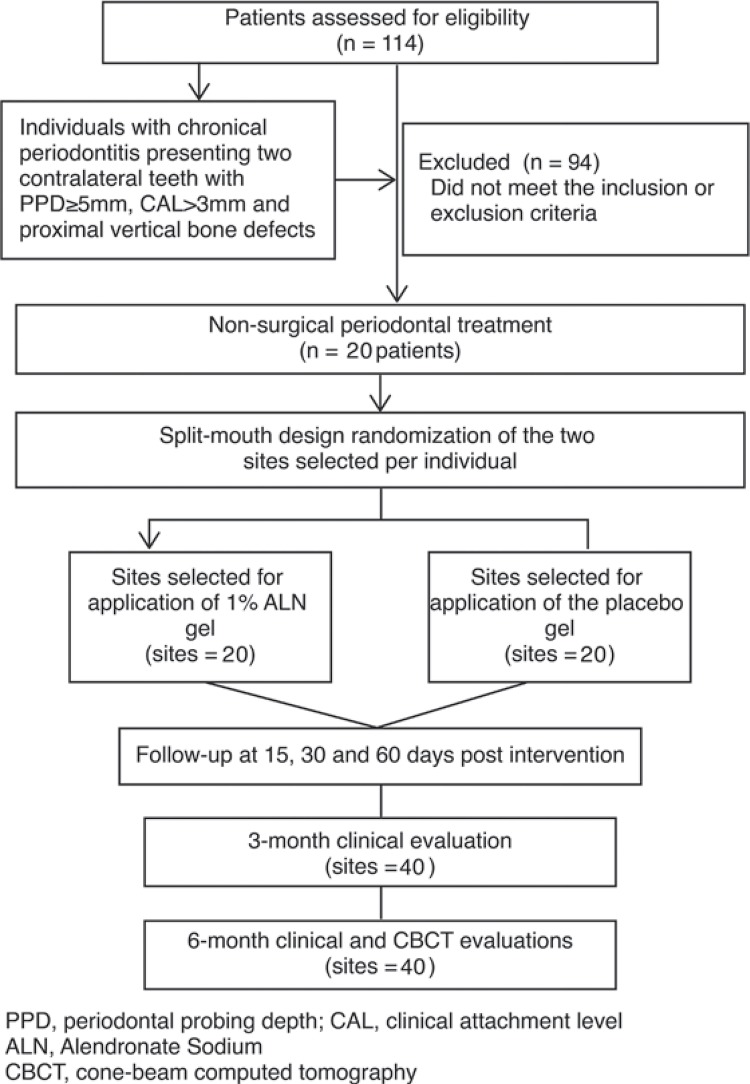
Study of the flowchart

A pilot study with five individuals was conducted with the purpose of training and adjusting study protocols. Data from this pilot study was not included in the final study. The study population of the present randomized, split-mouth, placebo-controlled clinical trial comprised patients looking for dental treatment between November 2012 and November 2013 (Figure 1). This study was approved by the Institutional Ethics Committee (protocol #22493714.5.0000.5149) and registered at Clinicaltrials.gov (NTC02470611).

The inclusion criteria were: a) moderate/advanced chronic periodontitis^3^, b) age between 35 and 60 years old, and c) presence of two contralateral teeth, featuring contact with the adjacent teeth, with proximal sites showing PPD ≥5 mm and CAL >3 mm, associated with proximal vertical bone defects, without furcation lesions, premature occlusal contact, or prostheses. In addition, these two contralateral teeth should have the same number of roots and be located on the same dental arch with <2 mm difference in their PPD baseline values.

The exclusion criteria were: a) antibiotics use or any other medication that could interfere in the results, b) periodontal treatment within the six months prior to the study, c) pregnancy or breastfeeding, d) depressed immune systems, e) diabetes or any other medical comorbidity that could impact on the general health, f) smoking, g) orthodontic appliances and/or removable prostheses, h) medical history of osteoporosis, and i) allergy to BPHs or history of their systemic use.

The sample size was calculated based on the primary outcome of the study, i.e., changes in CAL measurements. The mean and standard deviation values of CAL gain from the previous studies^28,29^were used as reference. Considering a 5% significance level, 80% power, and 25% difference between the studied groups, a minimum sample of 15 sites *per* group was determined to be necessary. Considering a loss of up to 30%, previously reported in similar studies, a sample number of 20 sites *per* group was defined as appropriate. Therefore, trough a split-mouth design, 20 individuals who met the inclusion and exclusion criteria were enrolled and the final sample was composed of 40 sample units: 20 sites allocated to 1% ALN gel application and 20 sites allocated to placebo gel application.

### Periodontal examination

The full-mouth PPD, CAL, BOP, and plaque index^30^ were evaluated at 4 sites *per* tooth (distal, mesial, buccal, and lingual) using a manual periodontal probe (PCPUNC-15, Hu-Friedy, Chicago, IL) at the baseline and 3- and 6-month follow-ups after treatment, by a single calibrated periodontist (P.A.D.O). The kappa values for the intra-examiner agreement of the PPD and CAL measurements at the baseline and 3- and 6-months follow-ups were >0.82.

### Radiographic and CBCT examination

At the baseline, the Kodak 2200 Intraoral X-Ray System X-ray machine (Eastman Kodak Company, Rochester, NY, USA) was used to acquire periapical radiographs of the teeth for diagnostic purposes. The Scan-X Duo system (Air Techniques, Melville, NY, USA) was used for digital photostimulable phosphor imaging. The radiographs were acquired following the paralleling technique by a single operator, using the Rinn (Dentisply, USA) positioning sensor model. The proximal vertical bone defects were defined according to the criteria adopted by Nibali, et al.^24^(2011).

The bone fill was evaluated at the baseline and at 6 months post-treatment using standardized tomographic images acquired through CBCT using the Kodak 9000 3D CBCT (Eastman Kodak Company, Rochester, NY, USA), operating at 60 kV and 10 mA, with an exposure time of 10.8 s, scanning at a collimation of 5.0 cm x 3.7 cm, and an isotropic voxel thickness 135 of 76x76x76 μm.

Two trained, blinded, and calibrated specialists (A.M.S.D.O and F.R.M) independently analyzed all tomographic images. The intra and inter-examiner weighted kappa values for the bone fill measurements, which were ≥0.95. The intra-class correlation test showed scores higher than 0.95. The tomographic images were measured directly using the Kodak Dental Imaging Software KDIS (Kodak Dental Systems, Rochester, NY, USA) on a computer with a GeForce 9500 GT graphics card, an LED monitor with 1920x1080 pixel resolution, and default brightness and contrast levels.

The electronic measuring tool included in the CBCT software allowed measurements to the nearest hundredth of a millimetre. The cross-sectional slice thickness was reconstructed to 1 mm for all measurements, and the location of each slice was identified using the location of the gutta-percha. All of the measurements were performed using only one computer, with unaltered screen settings. The examiners were allowed to modify bone density and image size to enable optimal viewing. The distance between the cement-enamel junction and the deepest point of the bone defect was used as the reference parameter for the analyzed measurements, as proposed by Misch, Yi and Sarment^21^(2006).

### Non-surgical periodontal therapy

After the baseline clinical and CBCT examinations, the individuals were provided with oral hygiene instructions and dental prophylaxis. Quadrant-wise supra and subgingival debridement were performed in weekly sessions by a trained and blinded periodontist (B.C.D). Scaling and root planing were performed under local anaesthesia using Gracey and McCall manual curettes (Hu-Friedy, Chicago, IL, USA). The curettes were sharpened before each use and discarded after six consecutive uses. For each individual included in the study, the sites selected for the subgingival application of either 1% ALN or placebo gel were scaled in the last session of the periodontal treatment. After scaling, the experimental periodontal sites were randomly assigned to receive either 1% ALN gel (Injecta Center, Ribeirão Preto, São Paulo, Brasil), according to the formulation proposed by Reddy, Kumar and Veena^27^(2005), or a sterile placebo (same chemical composition without addition of ALN) gel (Injecta Center, Ribeirão Preto, São Paulo, Brasil), by a blinded researcher (B.C.D). To maintain the patients’ and researchers’ blinding, the ALN and placebo gels were dispensed in identical syringes labelled as gel A or gel B, without any additional information except expiration date.

No antibiotics or anti-inflammatory agents were prescribed after periodontal treatment. Additional oral hygiene instructions and oral prophylaxis were periodically administered (15, 30, and 60 days after intervention). Any adverse effects were recorded at the recall visits.

### Statistical analysis

Periodontal clinical parameters were compared over time using the Friedman test. The Wilcoxon test was used to evaluate both the effects of each treatment on the CBCT measurements and the inter-group differences. The statistical analyses were performed using the statistical software SPSS (SPSS, Chicago, IL, USA) version 14.0 for Windows. Results were considered significant at probabilities lower than 5% (*p*<0.05).

## Results

The present study included 20 participants (mean age 44.9 years old), the majority of whom were female (60%), whose proximal contralateral sites (n=40) were analyzed. The majority of the sites selected for the topical application of either 1% ALN gel or a placebo gel were located on the lower posterior teeth (90%) (Table 1).

**Table 1:** Characterization of the sample according to the demographic variables and analyzed teeth (n=20 individuals)

Variables		N	%
Age (mean±SD)		44.9±7.9	
Gender	Female Male	12 8	60.00% 40.00%
Education level	< 8 years 8 to 12 years > 12 years	5 11 4	25.00% 55.00% 20.00%
Arch	Mandible Maxilla	36 4	90.00% 10.00%
Segment of the arch	Anterior Posterior	4 36	10.00% 90.00%

The periodontal treatment produced improvements in the clinical parameters of the participants (Table 2). The percentages of both BOP and PPD (4–6 mm and >6 mm) showed significant reductions after the periodontal treatment. On the other hand, a gain in CAL and an increase in the percentage of sites with PPD <4 mm were also observed over time.

**Table 2:** Full-mouth periodontal clinical parameters over time (n=20 individuals; n=523 teeth; and n=2092 sites)

Parameters	Experimental times			P-value
	Baseline	3 months	6 months	
Plaque index (mean±sd)	42.5±14.6	28.2±14.4	23.0±8.4	< 0.001 [Baseline > (3 months = 6 months)]
BOP (mean±sd)	34.5±25.0	16.7±11.7	14.5±9.8	0.003 (Baseline > 3 months > 6 months)
PPD (mm) (mean±sd)	3.2±0.7	2.5±0.6	2.3±0.8	< 0.001 (Baseline > 3 months > 6 months)
CAL mm (mean±sd)	2.6±0.9	2.1±0.9	2.1±0.9	0.001 [Baseline > (3 months = 6 months)]
% sites with PPD <4 mm	64.8±15.5	81.4±12.7	86.2±10.8	< 0.001 [Baseline < (3 months = 6 months)]
% sites with PPD between 4 and 6 mm	28.0±13.5	16.8±11.6	11.7±8.4	< 0.001 (Baseline > 3 months > 6 months)
% sites with	7.2±4.7	1.7±2.2	2.1±3.2	< 0.001
PPD >6 mm				[Baseline > (3 months = 6 months)]
% sites with CAL >3 mm	42.2±1.4	28.0±21.2	28.2±18.0	< 0.001 [Baseline > (3 months = 6 months)]

sd, standard deviation; p-value was calculated using the Friedman test; Significant p-values are indicated in bold. BOP= bleeding on probing; PPD= periodontal probing depth; CAL= clinical attachment level

Table 3 shows the comparative results of the primary outcome (CAL measurement) of the two treatment groups (1% ALN and placebo gels). Although the PPD reductions in both treatments at the 6-month follow-up were similar, ALN application after scaling produced a greater CAL gain at the 3-month follow-up. This difference was also observed at the 6-month follow-up.

**Table 3:** Comparison of the mean periodontal probing depth (mm) and clinical attachment level (mm) according to the treatment groups (1% ALN gel or placebo gel) over time

Parameters		Groups	Experimental times		p-value
		Baseline	3 months	6 months	
Periodontal Probing Depth (mm; mean±sd) (n=20 sites)	ALN	6.4±1.4	4.3±1.6	4.3±1.6	< 0.001 [Baseline > (3 months = 6 months)]
Placebo	6.2±1.6	4.4±1.8	4.4±1.7	0.004 [Baseline > (3 months = 6 months)]
p-value	0.468 (ALN = Placebo)	0.239 (ALN = Placebo)	0.127 (ALN = Placebo)	
Clinical attachment level (mm; mean±sd)(n=20 sites)	ALN	6.9±1.2	4.3±1.1	3.6±0.7	< 0.001 (Baseline > 3 months > 6 months)
	Placebo	6.7±1.1	5.1±0.9	4.3±0.7	< 0.001 (Baseline > 3 months > 6 months)
	p-value	0.356 (ALN = Placebo)	0.010 (ALN = Placebo)	0.021 (ALN = Placebo)	

ALN, Alendronate Sodium. sd, standard deviation. Significance probability was calculated using the Friedman test for comparison of the variables between the evaluation times and the Wilcoxon test between the treatment groups. Significant p-values are indicated in bold.

Bone measurements are shown in Table 4. The intra-group analysis revealed significant differences in the CBCT-based measurements only in the 1% ALN group (baseline, 4.5 mm; 6 months, 3.8 mm; *p*<0.003). However, the inter-group analysis revealed no differences between the mean measurements of the ALN and placebo gel-treatment groups (*p*=0.112).

**Table 4:** CBCT-based measurements of bone fill according to the treatment groups over time

Groups	Experimental times		p-value
	Baseline (mm)	6 months (mm)	
ALN (n=20 sites)	4.5±1.8	3.8±2.0	0.003 (Baseline > 6 months)
			
Placebo (n=20 sites)	5.1±2.2	4.9±2.0	0.099 (Baseline = 6 months)
p-value	0.470 (ALN = Placebo)	0.112 (ALN = Placebo)	

ALN= Alendronate Sodium. CBCT= cone-beam computed tomography. Significance probability was calculated using the Wilcoxon test for comparison of the variables between the groups and experimental times. Significant p-values are indicated in bold.

Both therapies were well tolerated by the participants. No periodontal abscesses occurred during the course of the study. Only one case of periapical abscess was observed; but the abscess was not in an experimental site and its treatment did not require antibiotics. None of the adverse effects was considered to be related to the investigational gel.

## Discussion

Few studies have focused on the topical application of ALN in humans^17,26-29.^ Despite the differences in methodology, these studies^25-28^ reported positive effects of ALN application in comparison to the placebo, which was evident from the reduction in PPD, gain in CAL, and filling of the vertical bone defects. Interestingly, the efficacy of 1% ALN in association with scaling and root planing in the treatment of infrabony defects was demonstrated even in patients with chronic^25^ and aggressive^29^periodontitis. Therefore, the present study was designed to investigate the efficacy of ALN as an adjuvant in the treatment of chronic periodontitis. Since CAL is considered the clinical gold standard outcome in clinical periodontology, it was selected as the primary outcome in the present study. The findings from the present study corroborate with the previously reported clinical efficacy of ALN^17,28,29^. Additionally, the results confirmed the efficacy of non-surgical mechanical procedures in the treatment of periodontitis.

In the present study, only the ALN group showed significant filling of bone defects, revealed by a decrease in bone height. Although ALN has been previously reported to stimulate the filling of periodontal defects, in the present study it showed no significant difference in comparison to the placebo. Similarly to the findings of a previous study^31^, ALN did not alter bone fill in the extraction sockets despite causing an enhancement in bone formation. However, we are not sure whether these changes could possibly be identified in periods of follow-up longer than 6 months. This limitation could be addressed in future studies. On the other hand, a positive aspect of the present study is the larger sample size compared with previous studies^27,30^, although study power was not higher than 80%. However, external validity is still a barrier to be overcome.

Additionally, it should be emphasized that ALN did not cause any local adverse events. This fact reinforces the advantages of its topical application over its systemic use, thus contributing to greater safety and better conformance of the individuals.

In the present study, the measurement of the interproximal bone defects was performed using the CBCT images acquired at the baseline and at 6 months post-periodontal treatment. A significant reduction in mean bone height was observed in sites that received 1% ALN gel, while the measurements of the sites treated with the placebo as with the adjunctive gel were unchanged. Some studies^27-29^have also reported a greater filling of bone defects with the application of the ALN gel. Interestingly, previous studies have used periapical radiographs, which have lower sensitivity than CBCT^21^. It is also relevant to mention that the improvements identified in the present study were associated with conservative procedures. Further studies are required to establish the pattern of clinical and tomographic outcomes exhibited upon the application of ALN in the surgical treatment of chronic periodontitis.

It is important to reinforce that a great debate exist regarding the use of systemic bisphosphonates and the incidence of osteonecrosis associated with surgical procedures in the oral cavity. There is some evidence suggesting that osteonecrosis can occur in individuals on long-term high potency bisphosphonates used via the intravenous route of administration^16^. Unfortunately, osteonecrosis of the jaw has been reported as an adverse event associated with the long-term or systemic use of BPhs^5^. The prevalence of this adverse event has been reported to be 0.5% and 1.8% in patients undergoing cancer treatment and on long-term intravenous medication, respectively; on the other hand, an average of 4.4 years of systemic use of BPhs in the treatment of osteopenia/osteoporosis resulted in osteonecrosis in a fewer number of patients (0.2%)^18^. For reasons not yet completely understood, oral osteonecrosis seems to have a few risk factors such as the changes in the patterns of bone resorption and remodeling, decrease in angiogenesis due to reduction in growth, migration and differentiation of endothelial cells, and the presence of inflammation/infection^4,18^.

Corroborating with our results, studies using low-dose topical ALN administration did not present adverse effects^17,26,27^. Nevertheless, caution should be exercised when considering the results of the present study in the clinical environment since future studies should be conducted on the potential cumulative effects of topical ALN administration.

Thus, topical 1% ALN might operate as a beneficial adjuvant in providing greater gain in CAL, although its application showed no significant differences when compared to the placebo in terms of the reduction of PPD and bone fill. In this sense, the present study can be considered a good starting point for future studies directed towards providing additional information on topical ALN use in periodontal therapy.

## Conclusions

The present study demonstrated that the topical application of 1% ALN improved the periodontal clinical status and might be a beneficial adjuvant to non-surgical periodontal therapy.
